# Recovery Based on Spirituality in Substance Abusers in Iran

**DOI:** 10.5539/gjhs.v6n6p154

**Published:** 2014-07-29

**Authors:** Abbas Shamsalinia, Kiyan Norouzi, Masoud Fallahi Khoshknab, Ali Farhoudian

**Affiliations:** 1 Department of Nursing, University of Social Welfare and Rehabilitation Sciences, Tehran, Iran; 2 Substance Abuse and Addiction Research Center, University of Social Welfare and Rehabilitation Sciences, Tehran, Iran

**Keywords:** recovery, substance abuse, spirituality, qualitative study

## Abstract

**Background and Purpose:**

Spirituality is an important factor influencing the decrease of substance abuse severity and maintenance of the recovery phase. This research, investigates the effect of spiritual experiences in the recovery of substance abusers.

**Material and Methods:**

Qualitative data was collected from 16 men and 6 women, selected through purposeful sampling to ensure an equilibrated gender representation and data from different recovery periods. Data were collected via semi-structured interviews.

**Results:**

Data showed two main categories: “Mutual relationship between spirituality and recovery,” divided into four subcategories: religious background, religious teachings, experience exchange, and support of family and society; and “A new perspective toward life” subdivided into access to calmness and spiritual development. A factor “spirituality meaning religion” arose repeatedly throughout the study.

**Conclusion:**

The results of this study can be useful for policy makers, care providers, families, and drug addicts. The promotion of spirituality in substance abusers can help in their struggle with temptation. Effective strategies to ensure drug abstinence and maintenance of the recovery phase are encouraging substance abusers and their families to participate in spirituality-based psychotherapy sessions held in addiction treatment centers, multi-disciplinary cooperation among the organizations involved in the addiction phenomenon, and training the families regarding the importance of spirituality in the mental health of their children through mass media.

## 1. Introduction

Substance abuse has become one of the most serious problems for human kind, representing one of the most complex human phenomena; no other phenomenon has ever challenged humans to this extent (Cavalcanti, 1994). Geographically located in a zone of transit and smuggling route for narcotic substances, Iran has become a hub for drug victims, one of the biggest worldwide (International Narcotics Control Board, 2008). The last nationwide study, in 2007 (RSA 2007), showed a total of 1.2 million substance abusers in Iran out of a population of 75 million people (1.6%) (Goodarzi et al., 2011); however, more pessimistic estimates suggest that this figure could reach 4,000,000 people (Mokri, 2002).

As a chronic disease (Arevalo, Prado, & Amaro, 2008; Saitz, Larson, LaBelle, Richardson, & Samet, 2008), addiction threatens the individual integrity, and decreases his/her control on substance abuse (Rutter, 2005). Many studies have been conducted to date on the prevention and treatment of drug addiction; however, few studies have focused on the recovery phase (Hser & Anglin, 2011). Recovery is a process that begins with refraining from substance abuse, and proceeds with a continued abstinence, followed by dramatic changes in the individual and his/her interpersonal relationships. Recovery requires getting back to normal life and rebuilding social relationships, including building or rebuilding individual, familiar, social, and spiritual relations. Recovery is a lifelong process involving several change stages, opening the individual possibility to recover his/her health and the type and level of improvement in his/her lifestyle (Dickens, 2011). There are several ways to recover (Belleau et al., 2007). Individual needs, attitude types, the degree of ability, and the behavior of each individual determine the personally unique recovery method (White, 2008). These methods are often strongly dependent on the social environment and grounded in cultural beliefs and traditions (Kaplan, 2008). One of the factors influencing recovery is spirituality. Previous research supports the positive role of spirituality in initiating the recovery phase and in ensuring a long-lasting recovery (Aldridge, 2000; Heinz et al., 2010; White, 2008). Because spirituality is rooted in beliefs; it can be an effective factor enhancing the recovery process; however, this aspect is often neglected; as a result, the probability of relapse increases (Heinz et al., 2010), Spirituality increases the potential to adapt, decreases the level of stress, and increases resilience before stress. It also leads to a purposeful life, and the reception of more social support among rehabilitating individuals (Wong & Yau, 2010). A strong spiritual and religious orientation increases the probability of recovery after substance abuse. Results from previous research show that a purposeful and meaningful life, spiritual activities such as praying, and participating in religious communities prevent high substance abuse at the time of recovery (Morjaria & Orford, 2002). Spirituality is a combination of experiences, thoughts, and behaviors. Spiritual thoughts include metaphysical or high beliefs; however, defining spiritual experiences is difficult due to its association with sentiment experiences, physiological sensations, and perceptions, which are all rooted in divinity or have high meanings or purposes (Arevalo et al., 2008; Lyons, 2012). Previous studies suggest that the degree of spiritual orientation and experience exert a powerful influence on the etiology, course, and outcome of substance use disorders. Many researches have supported the fundamental influence of spiritual orientation and experiences on the etiology of substance abuse, the course period, and its consequences (White, 2008); however, only few studies have focused on the role of spirituality in the recovery phase (Clark, 2012; Duffy & Blustein, 2005; Wright, 2003). Results from studies on recovery after substance abuse and the role of different factors during recovery highlight the need to consider emphasized different aspects of this phenomenon including spirituality. Despite accepting the importance of quantitative studies, it seems that the role of spirituality in the recovery of substance abusers has not been appropriately assessed so far in Iran, and the mechanism and role of spirituality in this regard remains unclear. Spirituality largely depends on the societal background and cultural aspects.

Results from previous studies focused on the problems and issues associated to the life of substance abusers raised the question on whether spirituality could influence their recovery and how they would explain its influence in their lifestyles. Set against this background, this study aimed to explain the role of spirituality in the recovery of substance abusers.

## 2. Materials and Methods

This study was conducted in 2012. A total of 22 Iranian nationals, aged 20 to 40 years of age, participated in this study, aimed to qualitatively analyze the role of spirituality in the recovery of substance abusers. All participants had a history of drugs abuse (opium, opium gum, morphine, heroine, hhashish, and Iranian crack), and had been through a minimum of 6 months of recovery. The study received the approval of the Ethics Committee of the University of Welfare and Rehabilitation Sciences (Tehran, Iran). All the participants received a full explanation of the research goals of the research units and provided written informed consent. After receiving their consent, data was gathered of substance abusers who fulfilled the inclusion criteria. Data were collected using semi-structured interviews. The participants were selected by purposeful sampling to ensure a gender-equilibrated sample and individuals from different recovery periods. The participants were interviewed including questions such as “Please speak about your experience in the beginning of substance abstinence.” Based on the participant´s answers, those were followed by probing questions to enrich the data. All interviews were conducted in private places, where patients were controlled through their period of recovery (e.g., Addiction treatment centers, The Association of Anonymous, or abstinence camps), in the cities of Ramsar and Tonekabon. Each interview was recorded using a digital recording device and lasted for 45–90 minutes. Data analysis and data collection were performed simultaneously. The data analysis was based on a conventional content analysis following the Graneheim and Lundman’s method (Graneheim & Lundman, 2004). Content analysis is a method to analyzed qualitative, abstracted, descriptive and interpretative data. It is used to identify the dominant and main themes, and is an appropriate method to investigate individuals’ experiences and attitudes in specific subjects (Sandelowski, 1995). All interviews were transcribed verbatim. These transcripts were read several times to achieve general understanding of the participants’ sayings. Subsequently, the meaning units, which included those sentences and paragraphs related to each other, were identified by their content and context, and the primary codes were extracted from them. These codes were then merged and categorized based on similarities and classified into subcategories representing different levels of abstraction. The subcategories were classified into categories based on at least one common feature, following the content analysis methodology (Graneheim & Lundman, 2004). Subjects within categories showed the highest similarity, while subjects among categories showed the highest inconsistency. The investigation, research results, methodology, data gathering and the study approval were checked by peers and members of the community. In this study, the transferability of the data was ensured through deep and analytic descriptions, the description of the study context, and in the clear description of challenges and limitations. Moreover, all research procedures, especially data analysis, were recorded in detail (Polit & Beck, 2010; Speziale, Streubert, & Carpenter, 2011). The participants were informed about the privacy issues and information, anonymity in all of the documents related to the research. They were also assured that they could withdraw from the research at any time.

## 3. Results

A total of 22 individuals (16 men and 6 women) participated in this study. Their average age was 30.7 ± 7.3 years. The most abused substance was opium (88.2 %) and the least abused was hashish (29.4%); however, most participants were abused of several substances simultaneously.

The average abuse period was 10.1 ± 9 years and the average recovery period was 3.6 ± 3 years. The two main categories extracted in this study were “*Mutual relationship between spirituality and recovery*” and “*A new perspective toward life*.”

Spirituality, in its religions meaning, repeatedly appeared throughout the data.

### 3.1 Mutual Relationship Between Spirituality and Recovery

Sspirituality strongly influenced the decision to initiate abstinence and the start of the recovery in most participants. On the other hand, the actual recovery influenced some participants to resort to spirituality. This research showed the category “Mutual relationship between spirituality and recovery”, as one of the main categories in this study. This category is in itself divided into four subcategories: religious background, religious teachings, experience exchange, and support from family and society. They are described below.

#### 3.1.1 Religious Background of the Patient and the Family

Most participants considered that having a religious background influenced their physical, mental, spiritual, and social maintenance. During hard times and under certain conditions, the patients felt that nobody could help them, but during periods of strength, the individual and families, religious values, and beliefs provided them with a background to consider God as the only way to salvation. Most participants agreed that in these situations, spirituality positively and strongly influenced the decision making for abstinence and beginning the way to recover:

“*I was crying all the time and prayed for the help of God because I was at the end of the line and I had resorted to everyone except God before that; thus, I found salvation in religion, and since my family were traditionally religious, I started attending religious ceremonies and practicing religion, specially praying*” (a 39-year-old woman, 6 years into recovery)

Based on their religious beliefs, most participants considered that feeling the presence of God was an important factor at the beginning and throughout the recovery process:

“*God liked me, so He chose me, and helped me, and saved me from the vortex of addiction*” (a 36-year-old man, 8 years into recovery).

The data showed a difference between men and women in this regard. For men, the religious beliefs and behaviors (prayers) were billed at the beginning of the road to recovery; however, after recovery initiated, the role of beliefs and resort to God, and being thankful to God, often diminished:

“*Once I overdosed, I was about to die. In that condition, I asked God to give me the opportunity to live once more and I promised Him not to abuse substances anymore. I said this and I just got up. Now, I sometimes say my prayers and I participate in religious ceremonies*” (a 30-year-old man, 2 years into recovery).

#### 3.1.2 Religious Teachings

An important finding of this study is that correct or incorrect teachings of religious concepts are suggested as an important factor influencing the decision making process to become drug-free:

“*In the funeral ceremony of my brother (who had died of drug overdose), I heard a sentence from a clergyman, who cared for me and influenced my decision for abstinence, and ethical, and spiritual changes through the recovery period*” (a 24-year-old man, 1 year into recovery).

#### 3.1.3 Experience Exchange

The participants in this study considered that the exchange of spiritual experience among themselves and their peers in abstinence centers was a factor influencing and leading to the growth of their spirituality.

“*I was fed mentally, psychologically, and spiritually within my peer community and I was placed in the spiritual path upon hearing their experiences of God’s helping them*” (a 32 year old woman, 7 years into recovery).

For some of the participants, the recovery itself was suggested as the main factor influencing their growing spirituality; the fear of God was replaced by the image of a loving God in the deeper layers of their hearts, and their internal pure religious beliefs revived and took a different pattern:

“*Before recovery, there was faith but it was due to fear of God, not to love for Him. But now in the recovery, it is my love for God that prevents me from doing bad things. That love started when I initiated drug abstinence*” (a 39-year-old woman, 6 years into recovery).

#### 3.1.4 Support From the Family and Society

The role of the family and addiction treatment centers in shaping the participants’ spirituality was also apparent. The following quotations describe this:

“*After drug abstinence, a lot changed in my life. Honestly, I owe all this to NA (Narcotic Anonymous) who revived me, and God in me, and now I feel proud that I became familiar with a community who played an important role in my change and growth*” (a 38-year-old man, 8 years into recovery).

For some participants, spiritual changes and the ability to stick to them depended on the constant participation in addiction treatment and their relationship with the addiction treatment centers:

“*The reason I am successful today is just that I constantly participate in recovery sessions, and I’m working on taking the 12 NA steps gradually*” (a 28-year-old man, 2 years into recovery).

### 3.2 A New Perspective Toward Life

Changing the perspective toward life and achieving a desirable life prospect was the second main category identified in this study, including the two subcategories “access to calmness” and “spiritual development”.

#### 3.2.1 Access to Calmness

Most participants in this study considered that resorting to God at the time of drug temptation was an important factor influencing their tranquility and in the struggling with temptation:

“*When I feel insecure and the temptation of substance abuse arises, I resort to God and He calms me. For example, before I go to bed at night, I thank to God for my abstinence today and I ask Him to help me again the rest of the days*” (a 25-year-old woman, 2 years into recovery).

#### 3.2.2 Spiritual Development

A number of participants stated that orientation to spirituality resulted in behavioral, emotional, and affective changes, represented by honesty, rectitude, and appreciation for God’s favors, sticking to immaterial values, and making their lives purposeful. According to the participants´ statements, they have taken important steps to make their lives meaningful, leading to a growing motivation to continue the recovery process. They regard recovery as a kind of freedom from the prison that substance abuse was, and also appreciate the independence that results from God´s help. As a consequence of this freedom, all participants could experience a real life:

“*Substance abuse is so strong that it enslaved me. It’d chained me like a prisoner, but now I really feel that I’m no longer in the captivity of drugs. Now, I take decisions by myself. It is mostly due to God’s help that I’m experiencing a real life*”. (a 25-year-old woman, 2 years into recovery).

Quitting from incorrect past behaviors, rebuilding their personality, and receiving respect from others create a spiritual feeling in recovering individuals, which helps them to continue with their recovery. All participants showed evident behavioral changes as signs of recovery. These changes give them a feeling of meaning and purpose in life. They felt satisfied and lively, and were very happy to acknowledge the newly acquired trust from society, to notice how much they changed, and to control and guide their instant wishes in a positive direction. Along with these changes, and the growing level of satisfaction, they regarded resorting to spirituality and performing religious behaviors as the most important factor: “*Because of honesty, rectitude, and pureness, as a result of my faith in God, that man (employer) trusted me and gradually the society came to me and I hoped that these behaviors were correct so I repeated them*” (a 40-year- old man, 7 years into recovery).

Spirituality in the religious sense

Most participants considered spirituality and religion as the same concepts:

“*Spirituality means to go to God and to bring God into your life; this makes you feel that you are not alone. Spirituality means praying and worshiping*”. (a 28-year-old-man, 1 year into recovery).

## 4. Discussion and Conclusions

Two main categories were identified in this study: “Mutual relationship between spirituality and recovery” and “A new perspective toward life.” With regard to the first category, the influence of the religious context, religious teachings, experience exchange, and the support from the family and the society is evident in this study. On the one hand, the participants of this study considered themselves to be at the end of the line, because of a sense of disappointment resulting from multitude rounds of abstinence, social seclusion associated to social stigma, contempt from others, and dysfunctional family relations. They believed that although you can use spirituality in any situation in life, it is often in critical life situations that you turn to it. Therefore, they regarded their feeling of loneliness, being at the end of line, and complete loss of reasons to live as the reasons to turn to spirituality. In order to escape from their permanent nightmare, these individuals tried and fail in different paths. At last, they conceived that in order to ensure abstinence and remaining at the recovery stage, they needed a force stronger than material ones. It is here that they resort to spirituality to be freed. In this context, factors like religious background of the individual and the family, religious teachings, experience exchange, and social supports are revealed as important. Similarly, a parallel effect observed in many participants into recovery was a return to intellectual and religious aspects that they did not observe during substance abuse. This finding is compatible with previously reported results^25^, suggesting that addiction requires a lifelong treatment (Blumberg, 2005; Kaplan, 2008). Therefore, to treat it requires a high consideration of spirituality and its growth as an effective factor influencing abstinence. This study, compatible with other studies, indicated that a positive religious context within the family, and religious teachings, play an evident role in the patient´s spirituality from the beginning of the recovery period and its continuity (Heinz et al., 2010). The role of the family in the creation and the development of spirituality and religious beliefs should also be considered. Acceptance of the drug addict as a human-being in the NA association, formation of an empathy sense and sharing common experiences have resulted in a sense of security in the participants of the present study. Consequently, they exchanged their experiences with their peers, especially those related to spirituality and its effects on their recovery. Besides the exchange of spiritual experiences, the work of the NA associations is also based in the use of divine methods for the patient´s recovery. The NA work is based on accepting and acknowledging the existence of an addicted person, the devastating results of addiction, and admitting and testifying for a high power (Green, Fullilove, & Fullilove, 1998). In these rehabilitation centers, the addicts receive, guidance based on the principles of spirituality, in the process of spiritual revival, to help them to foster a positive meaning in life. These principles include belief and surrender to a higher power, search for advancement in an intended relationship with the higher power through prayers, meditation, and the use of the individual´s spiritual awareness to transfer the message of recovery to those addicts still suffering from addiction (Miller, 1998). Therefore, given the positive influence of NA in abstinence from an addiction, and in helping maintaining abstinence, it is suggested that other organizations and families linked to drug addicts are considered more important for the drug addict than previously thought. However, another important finding of this study is that substance abuse seems to be often replaced by dependence on these centers, which can also be threatening to the health of these patients. Friedman-gel (2006) argues that, although the NA aims to promote mental accountability and individual independence in the addicts, sometimes this association performs in such a way that people tend to lose the control of their lives and, because of this belief, they do not take any actions to ensure their recovery. He recommends that self-control and self-recovery must be taken into account during recovery (Friedman-Gell, 2006). Here, we found a considerable difference between men and women in terms of time dedicated and tendency to spirituality, and their spiritual commitment in the way to recovery. Concentration on spirituality in men was especially strong when the individual faced a severe stressful situation or death of a close friend or relative. The data showed that men mainly turned to spirituality to save them from the vortex of addiction, but just when they found themselves at the end of a line or when they found nothing to stick onto. This finding is compatible with previous studies (Heinz et al., 2010). In contrast, women did not show this behavior. In addition, commitment to religious beliefs, spiritual commitment, praying and worshiping was less pronounced in men than in women. In women, spirituality was strongly related to the decrease in substance abuse, depression, anti-social behaviors, and suicide, and also to an improvement in the treatment results, high levels of life contend, and general welfare. In this group, the trace of faith and religious beliefs can be found under the help of God, talking to God, and praying in all recovery stages. Perhaps this difference can be attributed to intrinsic characteristics of the Iran’s society. In fact, the mental needs and resort to religion and spirituality were more evident in women and their tendency to religion and spirituality was deeper and more continuous than that in men. This finding follows previously reported results (Morjaria & Orford, 2002; White & Laudet, 2006). In this study, women were thankful to God for His favors and kindness in their process of recovery; however, men were less driven by the relationship between recovery and God’s favor. This finding follows the results reported by Mohseni (Mohseni, 2003).

In this study, the religious matters were more prominent in women than in men, even at the time of substance abuse. The results of Wright (Amini et al.), from a study conducted in the Unites States, showed that although women were aware of the existence of God during the period of substance abuse, they did not have any inclination to God or to pray until they reached the end of the line (the utmost misfortune and hardship) (Wright, 2003). In contrast, our findings show that women prayed, and resorted to the infallible Imams even during the substance abuse period. This can be due to the role of Iranian families in the formation of religious tendencies in daughters from the early childhood, for example, the mothers take their daughters to religious ceremonies with them. With regard to the reasons for the differences found between men and women in relation to the time dedicated and tendency to spirituality, and spiritual commitment during recovery, this might be due to different mentalities. The tendency to spirituality relates to the tendency to have a resort and a refuge. Women, especially during hard times, may tend to get involved in religious behaviors and to have spiritual tendencies for security; however, men might not show such strong tendency. On the other hand, the difference in spiritual needs between men and women might be the reason why this occurs. Spiritual support from the family and the society was shown to be one of the most important factors influencing the tendency toward spirituality. This finding is compatible with the results of other studies. A recovered individual enjoys a positive relationship with his/her family and social network, which provides financial, mental, emotional, and spiritual supports, as well as friendship, kindness, and love (Chen, 2006; Anderson, 2009). The recovered individual also believes that the religious beliefs of the family help to provide a meaning to different life situations through sharing the religious beliefs with the family members. In fact, the family fosters its members via a positive and promising perspective and by the provision of high and spiritual intentions and values (Andeson, 2009). A new perspective toward life is another main category in this study. This category is divided into two subcategories: “access to calmness” and “spiritual development”. The representations of spiritual development, such as honesty, rectitude, attracting societal confidence, sticking to recovery, and access to internal tranquility are among the main consequences of spiritual tendencies and religious beliefs in the participants of this study. This finding is compatible with the results of other studies (Aldridge, 2000; Heinz et al., 2010; Morjaria & Orford, 2002; White, 2008; Wong & Yau, 2010). Wong (2010) argues that through the increase of internal abilities, spirituality helps people to find meaning in their lives in cases of severe stress. Spirituality increases the ability to adapt, decreases stress, enhances resilience before stress, generates a purposeful life, and results in receiving more social support by the individual in recovery (Wong & Yau, 2010). Several previous studies showed that having a purpose and a meaning in life, spiritual activities like prayer and saying prayers, meditation, and taking part in religious ceremonies prevents from severe substance abuse in individuals in the road of recovery (Morjaria & Orford, 2002). In this study, the terms spirituality and religion considerably overlapped. Given the religious context of the Iranian society, this could be expected. A previous study suggested that in Islam, religion and spirituality are not separate concepts (Nir et al., 2013). Shehan (2005) also argued that there can be an overlap between spirituality and religion. Many people believe that they are spiritual but not religious, probably meaning that their sense of spirituality does not depend on a series of formal religious beliefs and activities. In any case, many people consider themselves to be both religious and spiritual (Sheehan, 2005). In a study by Lyons (2012) it is mentioned that the mental nature of spirituality indicates that everything in life can be spiritual, from participation in religious ceremonies, meditation, and performing daily affairs. Furthermore, he suggests that the primary difference between religion and spirituality is that spirituality is individual, but religion is mostly social (Lyons, 2012); however, a number of studies indicate that the concepts of spirituality and religion are often ambiguous or mistaken precisely due to their overlap. According to these studies, spirituality is a broader and a more comprehensive concept than religion, and it also incorporates religion (Bregman, 2006; Clark, 2012; Mooney & Timmins, 2007; Wong & Yau, 2010; Zwingmann, Klein, & Büssing, 2011). The factors that influence these interpretations include the events that had happened in the life of the individual, religious contexts, religious activities, gender, culture, temper, and the life stage (Sheehan, 2005).

In this study, the pace and tendency for spirituality, as well as resorting to religion can be found even on the first steps to decide for abstinence and in all recovery stages. In this regard, spiritual support from family and society, and the religious background of the individual are more prominent than other factors. Therefore, the promotion of spirituality in both family and society is the key to success in the recovery of the substance abuser. Since turning to spirituality and sticking with it are strategies for struggling against the temptation for substance abuse, and for maintaining abstinence after treatment, the enhancement of spiritual health and paying attention to it should be the first step in in the recovery process. The use of a multi-disciplinary collaboration, emphasizing spirituality can guarantee a very successful way to recovery, through learning to live without drugs and getting rid of the effects of the drugs in their lives. On the other hand, since one of the intra-personal aspects of mental health is religious beliefs and spirituality, which promotes mental health by influencing conduct and lifestyle, and because one of the main goals in recovery is access to mental health, this phenomenon should be increasingly considered in the recovery process. Teaching the families on the importance of spirituality in children´s health and the way to ensure their tendency for religion and spirituality from early childhood by mass media can provide a secure resort to religion in the future.

Encouraging the drug addicts and their families to participate in psychotherapy sessions based on spirituality, commonly held in addiction treatment centers, would enhance their tendency to participate in medical programs and continuing that, to change their attitude toward addiction, and repair their personality, intellectual, and religious defects, their mental tendency to abstinence from drugs. Ultimately, this is the main factor successfully leading to quit substance abuse and a permanent pureness. Multidisciplinary collaboration between organizations and associations engaged with the phenomenon of addiction, with a successful track record of activities and experience in this area, is an effective strategy to ensure abstinence and maintaining the recovery period.
